# Retraction: Overexpression of YAP and TAZ Is an Independent Predictor of Prognosis in Colorectal Cancer and Related to the Proliferation and Metastasis of Colon Cancer Cells

**DOI:** 10.1371/journal.pone.0281614

**Published:** 2023-02-07

**Authors:** The PLOS ONE Editors

Following the publication of this article [1], concerns were raised regarding results presented in Fig 5. Specifically,

- The following Fig 5A panels appear to partially overlap despite being used to represent different experimental conditions:
  ○ Parental, si-Con, and si-YAP
  ○ si-Con and si-TAZ
- The Fig 5C Parental and si-Con panels partially overlap despite being used to represent different experimental conditions.

The first author confirmed that the Fig 5A and Fig 5C panels originated from the same samples and explained that the partial overlap was a result of a misunderstanding in the labelling of the image data files. They provided underlying data and an updated figure. However, upon editorial assessment of the updated Fig 5, additional image overlap was detected for panels used to represent different experimental conditions. Similarly, editorial assessment of the underlying data identified multiple images that appeared to partially overlap, including images presented for different experimental conditions.

In light of the concerns affecting multiple figure panels and the corresponding underlying data that question the reliability and integrity of these results, the *PLOS ONE* Editors retract this article.

LW did not agree with the retraction. SS, ZG, XZ, SH, AY, WW, and QZ either did not respond directly or could not be reached.
